# Morphological and genetic evidence for a new karst specialist lizard from New Guinea (*Cyrtodactylus*: Gekkonidae)

**DOI:** 10.1098/rsos.170781

**Published:** 2017-11-15

**Authors:** Stuart V. Nielsen, Paul M. Oliver

**Affiliations:** 1 Department of Biological Sciences, Marquette University, Milwaukee, WI 53211, USA; 2 Ecology and Evolution, Research School of Biology, The Australian National University, Canberra, Australian Capital Territory 0200, Australia

**Keywords:** *Cyrtodactylus*, ecological diversity, gecko, morphometric analysis, specialization

## Abstract

Exposed limestone karst landscapes, especially in the tropics, are often home to distinctive and specialized biotas. Among vertebrates, a particularly large number of karst-associated lizard taxa have been described, but for the vast majority, evidence of specific adaptions to karst is lacking. A number of studies, however, have provided evidence of consistent morphological trends in lizards that use complex, three-dimensional, saxicoline habitats such as those that typify karst areas. Here we combine morphological and genetic data to test whether a newly discovered gecko from an extremely rugged karst area in New Guinea shows morphological trends matching those observed in other lizards associated with complex rock habitats such as karst and caves. Consistent with predictions, the new species' head is flatter and narrower than similar-sized relatives, and it has proportionally larger eyes and longer limbs. These trends indicate this taxon represents the second documented instance of karst specialization in a New Guinean vertebrate, and suggest morphological traits to test for evidence of specialized ecological associations in the many karst-associated *Cyrtodactylus* taxa from Southeast Asia.

## 1. Introduction

Large areas of exposed limestone karst in tropical regions present spectacular landscapes, and are home to unique, endemic and putatively specialized local biotas in Madagascar [1], Southeast Asia [2] and Australia [3,4]. Lizards often show a particularly close association with karst landforms, including numerous taxa that have only been described recently [5–10]. Two broad, and by no means mutually exclusive, hypotheses can be advanced to explain this association. First, refugial evolutionary dynamics, such as persistence through climatic change in microrefugia, was the driver of localized distributions [4]. Or second, adaptive processes led formerly more generalist lineages to become ecological specialists, restricted to the topographically complex, nutrient-poor and biologically distinctive habitats provided by karst [2,11].

Understanding patterns of morphological differentiation is one element of teasing apart the relative roles of these two processes. Morphological studies have suggested a suite of specializations occur in many lizards that use complex, three-dimensional rock structures, including, but not exclusive to karst [12]. These include relatively long limbs, a more gracile body, and often a flatter head [12–15]. Other studies have also suggested a tendency for highly specialized rock-dwelling lizards to have proportionally larger eyes [16], but this has not been tested systematically. In contrast, if taxa endemic to karst show little evidence of morphological differentiation, and are allopatric isolates separated from relatives by climatically unsuitable regions, it suggests that karst endemism may be more strongly linked to refugial than adaptive processes.

The bent-toed geckos (Gekkonidae: *Cyrtodactylus*) are the most speciose genus of geckos, occurring across India, Indochina, Southeast Asia and Australia [17]. Over 60 species from Indochina, Peninsular Malaysia and Borneo appear to be restricted to isolated karst regions, surrounded by unsuitable habitat for *Cyrtodactylus* or forest areas occupied by other congeners [10,18–21]. Despite this diversity and probable ecological specialization, there has been relatively little work on patterns of ecomorphological evolution in *Cyrtodactylus*. Recent work, however, that compared some ‘cave’ taxa (not exclusive to limestone) to more generalized relatives found them to have a gracile build, longer limbs and a dorsal colour pattern consisting of well-defined, narrow transverse bands [15,17,22].

Although most *Cyrtodactylus* diversity is concentrated in Asia, New Guinea, northeastern Australia and the surrounding islands of western Melanesia are also home to a monophyletic radiation of around 30 species, notable for the apparently parallel evolution of giant species [23]. While five Australian taxa within this radiation seem to be closely associated with saxicoline habitats [19], karst-associated *Cyrtodactylus* have not been reported from New Guinea. This is despite the presence of large areas of exposed limestone karst landforms [24,25]. Among other taxa, two widespread but patchily distributed bird and mammal species may be linked with New Guinea karst [26,27], and recent surveys note a distinctive plant fauna (characterized by the absence of some typically common taxa, some specialist forms and some elevational depression) [28]. However, compared to nearby regions in Southeast Asia, there has been little investigation into patterns of biotic endemism and specialization in the New Guinea karsts.

The Southern Fold Mountains are the largest tract of karst in Melanesia, extending along the southern edge of the Central Cordillera of New Guinea from Lake Kutubu in the east to the Star Mountains in the west [25]. Rainfall in this region is extremely high (up to 8 m per year) [29]. Consequently, limestone is both highly dissected and masked by vegetation [28], making the area difficult to access and thus poorly known. One of us (P.M.O.) recently surveyed the herpetofaunal biodiversity of an extremely rugged area of limestone in these ranges. During this survey, a novel and distinctive, slender-bodied, medium-sized (snout--vent length (SVL) 97 mm) species of *Cyrtodactylus* was collected. This species was abundant at the type locality on both low vegetation and karst structures, but has not been detected at nearby sites which lack exposed karst. Here we test if, relative to similar-sized congeners, this species shows evidence of the morphological specializations typical of lizards associated with complex, three-dimensional saxicoline microhabitats—specifically long limbs, a flattened head and/or disproportionately large eyes [12,13,16]. We also present a formal scientific description of the new species.

## 2. Material and methods

### 2.1. Material

New specimens were collected by hand at night while spotlighting between 19 April and 8 May 2013, humanely field-euthanized using standard practices (intraperitoneal and cardiac injection of MS-222) [30], preserved in formalin, and stored in ethanol. Types have been lodged at Museum Victoria or the South Australian Museum, and two additional samples are to be repatriated to the Papua New Guinea National Museum. Liver samples for genetic analysis were taken from all samples [30]. Genetic samples and comparative material (electronic supplementary material, appendices S1 and S2) used in this study are primarily stored at the following institutions: Australian Museum (AMS)—Sydney; Museum Victoria (NMV)—Melbourne; South Australian Museum/Australian Biological Tissues Collections (SAMA/ABTC)—Adelaide; Museum of Comparative Zoology (MCZ)—Harvard University, Cambridge; and Museum Zoologicum Bogoriense (MZB)—Bogor. Additional comparative data for taxonomic descriptions were taken from published literature [31–36] while most genetic data were also taken from recent publications [17,21,23].

### 2.2. Genetics

To ensure maximal compatibility with published sequence data for *Cyrtodactylus*, we generated new sequence data from the NADH dehydrogenase subunit 2 (*ND2*) for four samples of the new species, in addition to two samples of *Cyrtodactylus capreoloides* collected sympatrically. Laboratory protocols largely followed Sistrom *et al*. [37] with *ND2* and partial flanking tRNAs amplified using the primers M112F (5′-AAGCTTTCGGGGCCCATACC-3′) and M1123R (5′-GCTTAATTAAAGTGTYTGAGTTGC-3′) designed in the flanking methionine and alanine tRNAs.

Our final genetic dataset included 107 out of approximately 250 nominal *Cyrtodactylus* species globally, and 22 out of 25 recognized species from Melanesia. To initially place the putative new species within a phylogenetic framework, we analysed 987 bp of *ND2* data aligned using the MUSCLE algorithm [38] using default settings as implemented in Geneious v. 6.0.5 [39], and subsequently checked for missense mutations and correct reading frames. Phylogenetic trees were estimated using standard maximum-likelihood (RAxML v.7.2.8) [40] analyses using the GTR model implemented on the CIPRES Web portal version 3.1 for online phylogenetic analysis.

We used BEAST v.1.8.0 [41] to visualize the evolution of karst-associated taxa across the radiation of *Cyrtodactylus*. We aligned genetic data (see above) from the new taxon with a four-gene dataset of *Cyrtodactylus* sequences synthesized from published literature (electronic supplementary material, appendix S1) [17,23,31,42]. This alignment included *ND2* for all taxa, plus three nuclear genes (*MXRA-5*, *RAG-1* and *Phos*) for a reduced number of taxa (electronic supplementary material, appendix S3). We used a three-partition strategy following previous analyses of Melanesian *Cyrtodactylus* [23] (mitochondrial first and second codons; mitochondrial thirds and nuclear genes) with all partitions given the GTR + G model. Topology and timeframes of divergence were estimated using the uncorrelated lognormal model, birth–death speciation prior for 20 million generations, sampling every 20 000 with the first 20% of trees discarded as burn-in, giving a total of 800 trees from which to estimate topological support. Dating calibrations followed those used elsewhere [23], with the addition of a broad normally distributed prior at the base of sampled *Cyrtodactylus* (mean 32, s.d. 5.0) derived from an analysis of multi-locus nuclear data containing the majority of recognized gecko genera and major lineages [4,43]. Habitat was coded as a simple binary state (karst versus not karst) and transition patterns were estimated using a simple all rates equal model (electronic supplementary material, appendices S4 and S5).

### 2.3. Morphology

The following morphometric measurements were taken with digital callipers to the nearest 0.1 mm, with bilateral measures recorded from the left side of the body: SVL, tail length (from the posterior edge of the vent to the tip of the tail (TL), total length of original portion of tail (OT), trunk length from posterior edge of axilla to anterior edge of groin with limbs held at right angles (TrK), maximum head width (HW), maximum head height (HH), head length from tip of snout to anterior margin of ear opening (HL), distance from posterior edge of naris to anterior edge of eye (EN) (used as a proxy for snout-length), transverse diameter of orbital (OrB), internarial distance (IN), transverse diameter of ear (EAR), forearm length from base of palm to outer edge of elbow flexed at 90° (FA), and hindlimb length from base of heel to outer edge of knee flexed at 90° (HDL).

For taxonomic comparisons the following meristic counts were also taken: left and right enlarged supralabials (SUPR), to both the midpoint of the eye and to the rictus; left and right infralabials to rictus (INFR); rows of enlarged dorsal tubercles between the ventrolateral folds **(**not including those on fold itself) at the midpoint of body (DTR); ventral scale rows in a transverse series between ventrolateral folds at midpoint of the body (VENT); the number of subdigital lamellae (LAM), including both the narrow (distal to the inflection of the digit; not including the ungual sheath) and wide lamellae (proximal to the inflection of the joint) under the first and fourth digits of the left manus and pes; precloacal (PREPOR) and femoral pores (FEMPOR) where present (males only); postcloacal tubercles/spurs (PCTUB); and number of dark-brown, semi-distinct dorsal bands that cross the midline between limb insertions (DB).

### 2.4. Multivariate and univariate statistical analyses

All statistical analyses were performed in R [44]. To test for potential morphological correlates of living on karst, we used size-corrected mensural data for the new taxon, compared against a suite of six, similarly sized (max SVL 82–109 mm) Melanesian *Cyrtodactylus* species, including all possible closest relatives (electronic supplementary material, appendix S6). Field observations suggest most of these taxa are arboreal and occur widely away from karst [31,36,45]. We focused on similar-sized species to reduce the potential for generalized non-allometric growth trajectories to confound analyses. In particular, many other Papuan *Cyrtodactylus* are very large geckos [23], raising the possibility that processes such as peramorphosis could confound analyses of relative body proportions [46].

All variables were log transformed. Probable juvenile (smaller than the smallest male with well-developed pores and/or testes) and highly contorted specimens were excluded from statistical analyses. Body, head and limb measurements were corrected for body size by regressing against principal component 1 from an initial principal components analysis (PCA), in order to account for possible non-allometric growth [47]. An initial PCA of the size-corrected body and HL data was used to determine population-level distinctiveness in multivariate space. We also visualized patterns of morphological variation across taxa in phylomorphospace [48] based on species means for size-corrected traits and a reduced ML phylogeny for the seven focal taxa.

To initially visualize patterns of univariate variation across species, we used simple boxplots of the size-corrected morphological data. Because a number of our focal taxa are only known from a small number of specimens (less than 10), we used non-parametric approaches to test for significant variation in traits. First, we used a non-parametric MANOVA (*adonis* function in R package vegan [49]) to test for differentiation across taxa based on a distance matrix calculated across 10 000 permutations. We then used the Kruskal–Wallis rank sum test (*Kruskal.test* function) to compare morphological differentiation in traits across species pairs *post hoc* (with the significance value set to *p *= 0.05).

## 3. Results

### 3.1. Genetics

**Figure 1.**
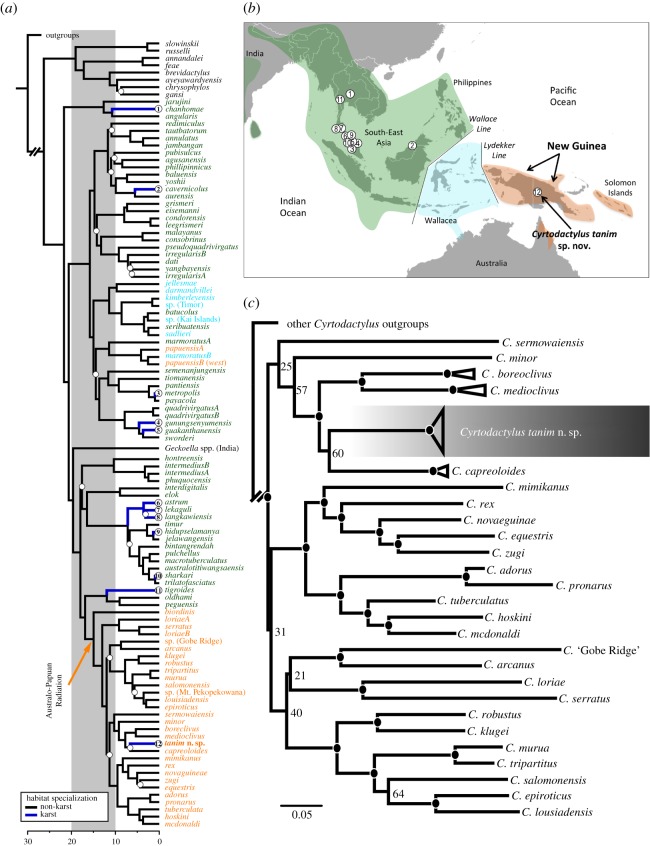
(*a*) Time-calibrated, multi-locus cladogram generated in BEAST reconstructing relationships within *Cyrtodactylus*. Tip names are coloured by area of occupation corresponding to the map in panel (*b*) (Asian—green, Wallacean—blue and Melanesian—orange). Branches coloured royal blue correspond to sampled karst taxa (with numbers in circles corresponding to localities in *b*). Posterior probability support values below a standard cut-off (PP < 0.90) are indicated with a white dot subtending the poorly supported node. (*c*) Maximum-likelihood RAxML phylogeny for the main Australo-Papuan *Cyrtodactylus* lineages estimated using approximately 900 bp of the mitochondrial *ND2* gene. Maximum-likelihood bootstrap support values (≥70%) are indicated by black circles subtending each node; when the value is below this threshold, it is given.

Phylogenetic analyses place the novel taxon within a strongly supported radiation of Australo-Papuan *Cyrtodactylus*, phylogenetically distinct (mid-Miocene or earlier splits) from all other sampled *Cyrtodactylus* inhabiting karst areas (figure 1*a*,*b*). Based on moderate statistical support (ML bootstrap 81%, Bayesian posterior probability 1.0), it is part of a recently identified clade of medium-sized, forest-dwelling species found in the mountains/hills of central and northern New Guinea (*Cyrtodactylus capreoloides, Cyrtodactylus boreoclivus* and *Cyrtodactylus medioclivus*) [31]. The new species is most closely allied to—but deeply divergent from—the more widespread and partially sympatric taxon, *C. capreoloides*, although this relationship was not strongly supported (ML bootstrap 60%, PP 0.88).

Based on our sampling of just under half of the recognized species of *Cyrtodactylus* (109 taxa or candidate taxa), at least nine independent transitions into karst microhabitats were inferred across New Guinea, Borneo, Peninsular Malaysia and Indochina. Ancestral states estimation indicated that the new species provides the first evidence of a shift into this habitat in Melanesia. No transitions out of karst, and just one potential example of speciation within karst, were inferred.

The new species is estimated to have diverged from its putative closest relative, *Cyrtodactylus capreoloides*, around the late Miocene (mean age estimates in millions of years ago 6.7, highest posterior distribution 5.1–8.2). Estimated divergence times of other karst lineages varied widely, ranging from mid-Miocene to Pliocene (15–1 million years ago) (figure 1*a*). However, as sampling in many parts of the wide range of *Cyrtodactylus* was very sparse and many potential sister lineages are missing, older divergence dates in particular are likely to be overestimates and should be treated with caution.

### 3.2. Morphology

**Figure 2.**
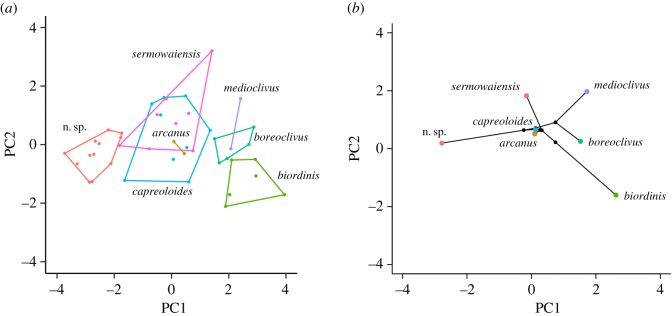
(*a*) PCA plots of size-corrected data for seven species of *Cyrtodactylus* from Melanesia. (*b*) Phylomorphospace plots of species means for PCA analyses superimposed on phylogenetic relationships for seven species of *Cyrtodactylus* from Melanesia.

Multivariate analyses of size-corrected data separated the novel species from all other taxa sampled on PC axis 1, with no overlap (figure 2*a*). All morphological characters except trunk length (TrK) were strongly loaded onto PC axis 1 (electronic supplementary material, appendix S7). Phylomorphospace plots also indicated that the new taxon was distinct from all the other medium-sized *Cyrtodactylus* in our sample from New Guinea along PC axis 1, including the three most closely related species *C. boreoclivus*, *C. capreoloides* and *C. medioclivus* (figure 2*b*).

**Figure 3.**
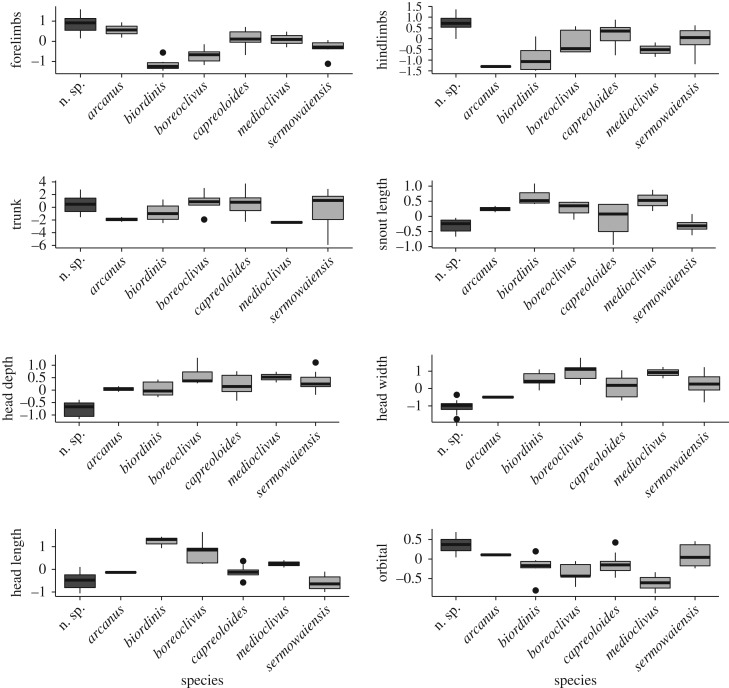
Boxplots of size-corrected values for eight mensural variables in a new species of *Cyrtodactylus* from Papua New Guinea karsts, and six other similar-sized (max SVL 80–120 mm) species of Melanesian *Cyrtodactylus*.

Visualization of univariate data indicated that the new species had a proportionately shallower and narrower head than all other sampled taxa, with very little overlap of values. Mean relative forelimb and hindlimb length, and orbital width were also the highest among sampled taxa, although the distribution of these values overlapped more considerably (figure 3).

Non-parametric MANOVA detected highly significant differences between taxa (*p *< 0.005). Subsequent *post hoc* non-parametric Kruskal–Wallis tests detected significant differences between taxa in all variables apart from trunk length (TrK). Of the 20 pairwise comparisons that significantly differed (with significance value set at 0.05), 18 involved the new limestone species. Transverse diameter of the orbital showed the strongest pattern, and was significantly wider in the new species than four of the six sampled taxa, including all three closest relatives. The new species also had a significantly shallower head than three other taxa (including two close relatives), and a narrower head than four other taxa (including two close relatives). While mean forelimb and hindlimb length was longer in the new species, in our statistical tests this did not differ significantly from most other species. Likewise, while head length and snout length showed some evidence of differentiation in univariate boxplots, most comparisons with other taxa were not statistically significant.

**Figure 4.**
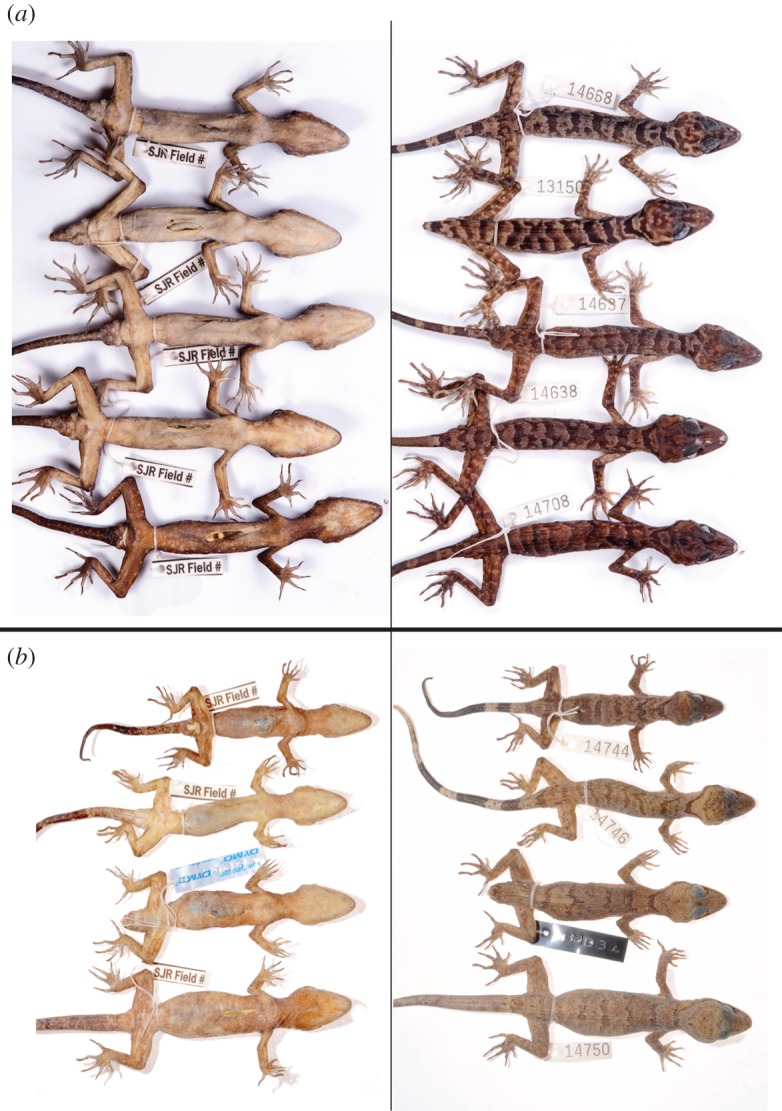
(*a*) Colour and pattern variation on the dorsal and ventral aspects of a subset of males of the type series of *Cyrtodactylus tanim* n. sp. (*b*) Colour and pattern variation on the dorsal and ventral aspects of a set of male *Cyrtodactylus capreoloides*, the probable sister taxon to *Cyrtodactylus tanim* n. sp.

In overall appearance, the new species appeared much more gracile than the related and sympatric *Cyrtodactylus capreoloides* (figure 4).

### 3.3. Systematics

The combination of genetic and morphological data confirms that these samples represent a novel taxon that is genetically divergent and morphologically distinctive from all other *Cyrtodactylus*, and that occurs in sympatry with its potential sister taxon *Cyrtodactylus capreoloides*. We here describe it as a new species.

***Cyrtodactylus* Gray, 1827**

**Figure 5.**
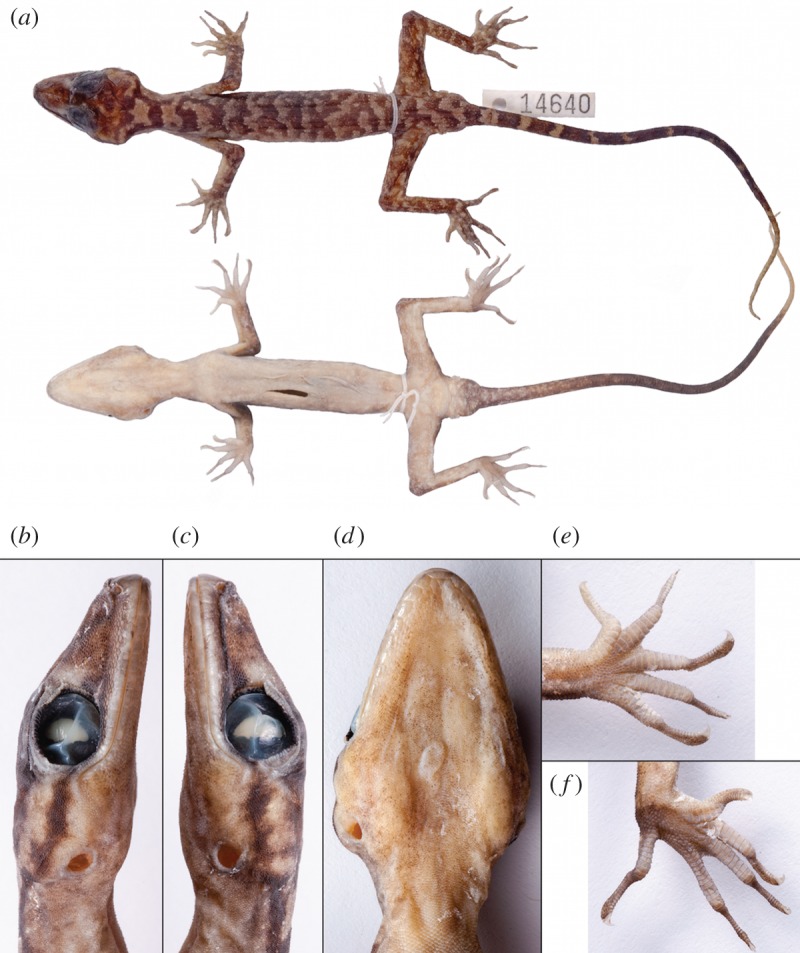
Holotype of *Cyrtodactylus tanim* n. sp. NMV75956, showing the dorsal and ventral aspects of the habitus (*a*), the right (*b*), left (*c*) and ventral (*d*) aspects of the head, and the plantar surfaces of the left manus (*e*) and pes (*f*).

**Figure 6.**
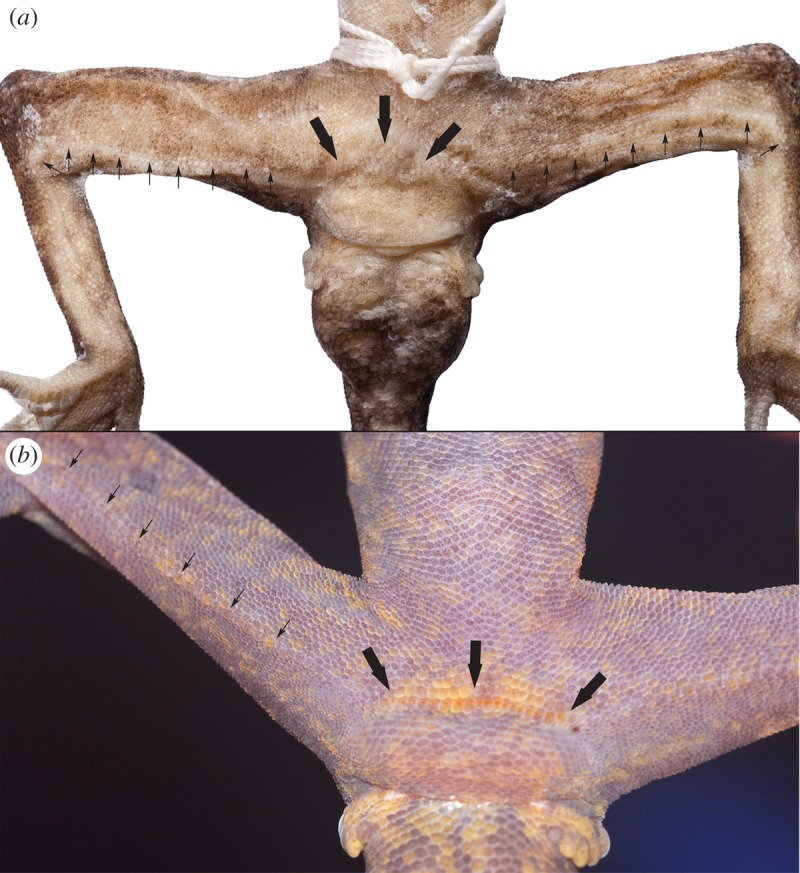
Pore series of the holotype of *Cyrtodactylus tanim* n. sp. NMV75956 (*a*), as well as a paratype NMV75962 (*b*) in life. Note the more visible femoral and precloacal pores in (*b*) and the diastema of slightly enlarged, but non pore-bearing scales between the femoral and precloacal series.

**Figure 7.**
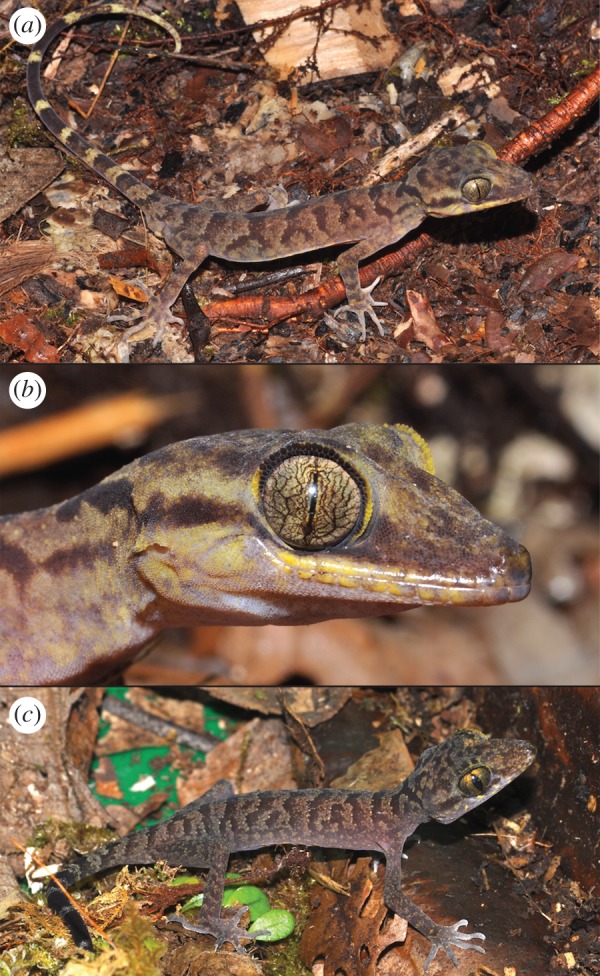
*Cyrtodactylus tanim* n. sp. in life. Paratypes SJR14637 (*a*,*b*) and NMV75961 (*c*) displaying juvenile coloration. Photographs: Paul M. Oliver.

*Cyrtodactylus tanim* n. sp. (figures 4–7).

#### 3.3.1. Zoobank LSIDs

Original publication: urn:lsid:zoobank.org:pub:422F5175-E543-43E1-A2A1-7602C2987B48

Species combination: urn:lsid:zoobank.org:act:0E895BC0-CF6C-43EE-9BE6-39F662DE083F

#### 3.3.2. Holotype

NMV75956 (F# SJR14640), adult male with original tail, Papua New Guinea, Western Province, limestone range approximately 9 km east of Kaiangibip Village, 1075 m.a.s.l. (5.53° S, 141.56° E) collected by P. M. Oliver (19 April 2013), with frozen tissue for genetic analysis at Museum Victoria.

#### 3.3.3. Paratypes

Papua New Guinea, Western Province (*n* = 12). NMVD75957 (SJR14638), SAMA R70319 (SJR14668), SJR14637, SJR14709 (males), NMVD75958–75960 (SJR14639, SJR14669, SJR140670) (females), NMVD75961 (SJR14717) (juvenile), all with same locality and collector details as holotype and collected between 19 and 20 April 2013; NMVD75962–63 (SJR14708, SJR14743), SAMA R70320 (SJR13150) (all males) Mt Uni, approximately 6 km west of Kaiangibip Village, approximately 540 m.a.s.l. (5.48° S, 141.54° E) collected between 26 April and 3 May 2013; NMVD75964 (PMO39) (male) approximately 9 km east of Kiangibip Village, approximately 760 m.a.s.l. (−05.51°, 141.57°) collected 8 May 2013.

#### 3.3.4. Diagnosis

*Cyrtodactylus tanim* n. sp. can be distinguished from all other Melanesian and (Wallacean) *Cyrtodactylus* by the following unique combination of characters: moderate size (SVL to 96.7 mm) and slender, with a relatively narrow head (HW/SVL 0.17–0.19), mid-dorsal tubercles in 14–16 longitudinal rows at midpoint of body, ventrolateral fold without enlarged tubercles, subcaudal scales not transversely widened, pores in a tripartite series, precloacal pores obvious and of moderate number (15–17), femoral pores minute and numerous (31–30 per limb, 66–76 total), and dorsal colour pattern on torso consisting of six to nine semi-distinctly defined, alternating dark-brown bands or blotches, on a medium-brown background.

#### 3.3.5. Description of the holotype

Adult male. Moderately large (SVL 96.7 mm) with slender habitus. Head large (HL/SVL 0.24), moderately slender (HW/SVL 0.17) and clearly distinct from neck (figure 5*a*). Snout longer than broad, rounded in dorsal profile and truncate in lateral profile (figure 5*b*,*c*). Eye to naris distance slightly greater than orbital diameter (EN/OrB 1.08), loreal region slightly inflated, interorbital region and top of snout concave, canthus rostralis rounded, weakly defined. Eyes quite large (OrB/HL 0.32), pupil vertical, supraciliaries extending from anteroventral to posterodorsal edge of orbit, longest at the anterodorsal margin. Ear opening rounded, bordered by distinct dorsal skin fold.

Rostral rectangular, wider than high, with medial suture extending ventrally approximately halfway from dorsal edge and terminating in short ventrolateral bifurcation, bordered dorsally by two flattened supranasals and one large and two small internasals. Nares bordered by first supralabial (point contact), rostral, 1 enlarged supranasal, and 5–6, granular postnasals. Supralabials generally wider than high, 11–12 total, 10 to midpoint of eye. Head, temporal and nuchal scales small and granular, interspersed with numerous enlarged weakly conical tubercles, approximately two to three times width of surrounding scales in temporal and occipital (and posterior nuchal) regions. Enlarged infralabials slightly to much wider than high, 14 on either side, bordered by rows of slightly enlarged scales that grade into small granular gular scales. Mental wider than long, broadly triangular, bearing distinct groove at contact with postmentals, in contact with first infralabials, and with a broad, lateral sulcus extending between the anterior tips of the two postmentals (figure 5*d*).

Body moderately gracile (TrK/SVL 0.47) with weak ventrolateral folds. Dorsum with approximately 15 rows (not including ventrolateral fold) of tubercles up to three to four times width of surrounding small, granular scales. Ventral scales much larger than dorsal scales, increasing in size medially, arranged in approximately 50 rows at midpoint of body. One semi-continuous row of enlarged, pore-bearing femoral scales per hindlimb, extending from the knee to near the groin in a series of 35–37 pores, with two additional pore-bearing scales parallel to the main row at the knee. Pore-bearing scales only slightly larger and weakly contrasting against rounded, weakly imbricate ventral femorals, but larger and distinct from granular posterior femorals. Precloacal pore row separated from femorals, in a distinct series of 16.

Limbs slender, forelimbs (FA/SVL 0.15) shorter and less robust than hindlimbs (HDL/SVL 0.19). Lateral and dorsal surfaces of crus with numerous conical tubercles, antebrachium with few and localized tubercles extending to the elbow. Digits long and well developed, inflected at basal interphalangeal joints (figure 5*e*,*f*); subdigital LAM smooth, rounded, undivided and expanded proximal to digital inflection (9–11–13–13–10 manus; 8–12–14–13–11 pes); narrow distal to digital inflection (9–10–12–13–12 manus; 9–11–13–14–13 pes) (counts excluding ungual sheath); large recurved claws sheathed by a dorsal and ventral scale.

Tail original (117 mm) and thin, scalation heterogeneous and irregular, grading from smaller dorsal scales to slightly larger scales ventrally. Hemipenile sacs swollen and prominent, each with four rounded cloacal spurs at anterolateral margin.

#### 3.3.6. Colour in ethanol

Dorsum consists of nine dark-brown, irregular, transverse bands or stars between shoulders and pelvis, interspersed by lighter greyish cream-coloured regions of approximately similar width. Dark-brown bands bordered by lighter, interspersed tubercles. Bands between the shoulder and occipital regions most distinct, but irregularly shaped. Nuchal band joins occipital band and extends as a stripe anterolaterally to the ventral orbital margin. Crown of head, supraorbital region and snout dark-brown, with occasionally lighter cream-coloured splotches with brown tinge. Supraciliaries dark-brown. Limbs and toes distinctly splotched to banded with dark-brown on medium-brown on upper and lateral surfaces. Ventral ground coloration cream with brownish tinge and extensive, lighter, off-white flecks throughout, generally composed of one to a few scales. Dorsal and lateral surfaces of tail darker chocolate brown with six indistinctly edged, dark-brown blotches or bands, separated by thin, creamish bands, before gradually lightening to cream again at the distal end. Subcaudal surface dark-brown with scattered lighter cream-coloured spots, becoming more immaculately dark-brown posteriorly, before gradually lightening at the tip.

#### 3.3.7. Details of holotype

Measurements (in mm): SVL 96.7; TL 117; TrK 45.9; HW 16.9; HH 9.5; HL 23.6; EN 8.2; IN 3.4; OrB 7.6; EAR 1.6; FA 14.8; HDL 17.9. Meristic data: SUPR 12R/11 L (10 to midpoint of eyes); INFR 14; INTER 2; DTR 15, VENT 50. LAMT1 8 expanded, 10 narrow; LAMT4 13 expanded, 15 narrow; POR 90 (PREPOR 16, FEMPOR 38R/36 L).

#### 3.3.8. Variation

Summary of meristic values for all adults (nine males, three females) in the type series are as follows (mean, with the range in parentheses): SVL 92.7 (88.0–96.7); TL 79.8 (13.0–117.0); OT 107.5 (95.0–117.0); TrK 44.8 (42.5–47.6); HW 16.6 (16.1–17.5); HL 23.6 (22.5–24.7); HH 9.3 (8.7–9.6); EN 8.3 (7.8–8.7); OrB 7.3 (6.9–7.7); EAR 1.5 (0.9–2.0); FA 15.1 (14.7–15.9); HDL 17.9 (16.9–18.5) (table 1 for a breakdown of measurements by sex).

**Table 1.** Variation in morphological characters of type series of *Cyrtodactylus tanim* n. sp.

	males (*n* = 9)		females (*n* = 3)		
	range	mean	range	mean	juvenile (*n* = 1) value
SVL	89.9–96.7	93.1	88.0–95.0	91.7	58.5
TL	74.0–117.0	101.5	105.0	—	79.0
OT	104.0–117.0	110.0	95.0	—	79.0
TrK	42.5–47.3	44.5	44.7–47.6	45.7	28.5
HW	16.1–16.9	16.4	16.2–17.5	17.1	10.7
HH	8.7–9.6	9.3	8.7–9.6	9.2	6.3
HL	22.5–24.4	23.6	22.8–24.7	23.6	15.9
EN	7.8–8.6	8.2	8.1–8.7	8.4	5.6
IN	3.0–3.5	3.3	3.3–3.5	3.4	2.4
OrB	6.9–7.7	7.3	7.0–7.6	7.3	5.1
EAR	0.9–2.0	1.5	1.2–1.8	1.5	0.7
FA	14.7–15.4	14.9	15.4–15.9	15.6	9.3
HDL	16.9–18.5	17.9	17.4–18.4	17.8	10.7

Summary mensural data for these same individuals are as follows in mm: SUPR (to midpoint of eye) 9.9 (9–11); SUPR (rictus of mouth) 12.1 (10–14); INFR 12.9 (11–14); LAMT1 expanded 8.4 (7–9), narrow 8.4 (7–10); LAMT4 expanded 12.3 (11–14), narrow 13.2 (11–15); DTR 15.2 (14–16); VENT 49.6 (46–54); POR 89.1 (83–94); PREPOR 16.1 (15–17); FEMPOR 36.5 (31–39) per hindlimb with 0–2 pore bearing scales out of line at the knee, PCTUB 3(1–4).

Dorsal colour pattern always consists of variably defined, contrasting transverse bands of dark to chocolate-brown against an off-white to cream-coloured to light-brown ground colour (figure 6). Dark bands fairly well-defined, particularly along their posterior borders; roughly equivalent width to or slightly narrower than the intervening lighter bands, both of which are less than half the width of the trunk. Occasionally spots invade the lighter bands. Some individuals have a lighter background coloration near the temples/occiput and light band along the supralabials. Venters cream to light-brown, often punctuated with lighter-coloured spots. Subcaudal surfaces variably spotted at the dorsal end to uniformly dark-brown, before gradually lightening at the tip.

All specimens have 14–16 tubercle rows at midbody. Subcaudal scales uniformly to slightly enlarged compared to those positioned dorsolaterally.

The colour pattern and scalation of the sole juvenile paratype (NMV75961, 58.5 mm SVL) is similar to that of the adults. Faint precloacal pores are present but difficult to count.

#### 3.3.9. Comparisons

*Cyrtodactylus tanim* n. sp. differs from Australasian *Cyrtodactylus* species in the *louisiadensis*, *loriae*, *novaeguineae* and *tuberculatus* groups in its smaller size (adult SVL <100 mm versus >100 mm), in having minute femoral pores (figure 7) much smaller than the precloacal pores (versus clearly visible and similar sized), in lacking enlarged tubercles on the ventrolateral folds (versus present), and in lacking enlarged subcaudal scales (versus present in the *C. louisiadensis* and *C. tuberculatus* groups only) [32,34,35]. *Cyrtodactylus tanim* n. sp. differs from *C. minor* and *C. papuensis* in its larger adult size (SVL <80 versus >80 mm), much higher total number of pores (<30 versus >60), and further differs from the latter in lacking a precloacal groove [34,50].

*Cyrtodactylus tanim* n. sp. differs from similar-sized (max adult SVL ∼ 80–110 mm) but distantly related Melanesian species as follows: from *C. aaroni* and *C. mimikanus* by the absence of enlarged subcaudals (versus present), dorsal pattern of moderately wide jagged dark-brown dorsal bands (versus very wide chocolate-brown bands separated by thin light stripes), and higher total number of pores in males (>80 versus <80) [51]; from *C. arcanus* by the absence of enlarged subcaudals (versus present) and longer legs (HDL/SVL <0.17 versus >0.18; table 2); from *C. biordinis* (Solomon Islands) by its higher number of dark transverse bands (6–8 versus 3–4), more numerous dark-brown blotches on the head and neck (versus two thick dark-brown postorbital stripes extending posterior to forelimbs, and a single large dark-brown spot); from *C. derongo* in smaller size (<97 versus >104 mm) and in having prominent dark-brown dorsal bands (versus unpatterned dorsum); from *C. nuaulu* (Seram Island) by lacking enlarged dentate tubercles on the tail (versus present), in lacking a precloacal groove (versus present) and its higher number of and darker dorsal bands (6–8 versus 3 greyish brown); and from *C. sermowaiensis* by the presence of enlarged femoral scales in both sexes (versus absent), the presence of precloacal and femoral pores in males (versus absent), and its longer limbs on average in proportion to SVL (FA/SVL 0.153–0.175 versus 0.138–0.159).

**Table 2.** Diagnostic characters separating *Cyrtodactylus tanim* n. sp. from similar-sized *Cyrtodactylus* species in mainland Papua New Guinea.

	*tanim* n. sp.	*arcanus*	*boreoclivus*	*capreoloides*	*medioclivus*
*n*=	12	2 (both F)	5	5	2
SVL (in mm)	89.9–96.7	84–92	104–109	62–84	97.2–103.4
TrL/SVL	0.463–0.516	0.441–0.458	0.368–0.496	0.442–0.484	0.449–0.452
TL/SVL	0.147–1.210	0.714–1.011	1.035–1.257	1.119–1.320	0.812–1.224
OT/SVL	0.146–1.210	0.179–0.772	0.199–1.257	0.209–1.158	0.097–1.019
FA/SVL	0.153–0.175	0.150–0.158	0.144–0.160	0.137–0.147	0.155–0.159
HDL/SVL	0.183–0.201	0.163–0.165	0.179–0.197	0.164–0.185	0.177–0.187
HW/SVL	0.172–0.190	0.179–0.180	0.193–0.213	0.171–0.197	0.197–0.203
HL/SVL	0.244–0.260	0.252–0.260	0.252–0.268	0.249–0.262	0.258–0.260
HD/SVL	0.096–0.105	0.105–0.111	0.108–0.119	0.106–0.118	0.110–0.115
EN/SVL	0.085–0.094	0.093–0.093	0.093–0.103	0.092–0.096	0.094–0.101
OrB/SVL	0.075–0.083	0.074–0.075	0.065–0.075	0.067–0.072	0.065–0.071
IN/SVL	0.031–0.039	0.039–0.041	0.037–0.044	0.038–0.041	0.036–0.040
EAR/SVL	0.010–0.022	0.020–0.021	0.014–0.023	0.009–0.107	0.015–0.023
SUPR (R) (to midpt. of eye/rictus)	9–11/10–14	8/13–14	7–9/9–11	5–9/9–12	8/10–11
SUPR (L) (to midpt. of eye/rictus)	9–11/10–14	8–9/12–14	7–9/9–12	7–10/10–12	8–9/10–11
INFR (R)	11–14	12	8–10	9–12	9–10
INFR (L)	12–14	11–12	8–10	9–13	10
DTR	14–16	22–25	16–19	20–22	20
VENT	46–54	37–40	36–44	31–39	43
INTER	1–4	5	?	1–3	0
POMEN	2	2	?	2–3	2
LAM first toe (expanded/narrow)	7–9/7–10	6–8/7	9–10/7–10	6–8/5–9	8/8–9
LAM fourth toe (expanded/narrow)	11–14/11–15	10/9–10	11–13/10–14	8–11/6–14	9–14/11–13
PREPORES	15–17	—	12	13–14	12–13
FEMPORES (R,L)	31–38,34–39	—	18–25,17–24	22,20	19–24,21
PATUB (R,L)	2–4,1–4	2,2	2,2	2,2	3,3

*Cyrtodactylus tanim* n. sp. differs from *C. boreoclivus* and *C. medioclivus* in lacking transversely enlarged subcaudal scales (versus present), by its smaller size (SVL <96.7 versus >97.2 mm), higher total number of femoral/precloacal pores (>83 versus <62), slightly more shallow head (max HD/SVL <0.105 versus >0.108) and lower number of dorsal tubercle rows (14–16 versus 20 [*C. medioclivus* only]) (table 2). It occurs in sympatry with its putative sister taxon, *C. capreoloides*, at lower elevations, but differs from this taxon in its larger size (adult SVL >89.9 versus <84.0 mm), much longer limbs (FA/SVL >0.153 versus <0.147, HDL/SVL 0.183–0.201 versus 0.164–0.185), fewer dorsal tubercle rows (14–16 versus 20–22), more shallow head depth (HD/SVL 0.096–0.105 versus 0.106–0.118), slightly shorter snout (EN/SVL 0.085–0.093 versus 0.092–0.096), wider orbital (OrB/SVL 0.075–0.083 versus 0.067–0.072) and dorsal pattern (6–8 dark-brown transverse bands versus 5–6 light, greyish brown bands) (table 2).

#### 3.3.10. Distribution and natural history

Currently known from three sites spanning an elevation from approximately 540 to 1075 m.a.s.l. in near-impenetrable limestone country just east of Kaiangabip Village, Western Province, Papua New Guinea. Similar limestone country is widespread, but difficult to access, along the southern edge of the Central Cordillera, and this species is likely to have a wider range than is currently known.

A similar, moderately sized *Cyrtodactylus* with many dark-brown dorsal bands was seen—but not collected—in limestone areas in the north of Gulf Province [52]. If this is also *Cyrtodactylus tanim* n. sp., then it will have a range spanning over 300 km.

**Figure 8.**
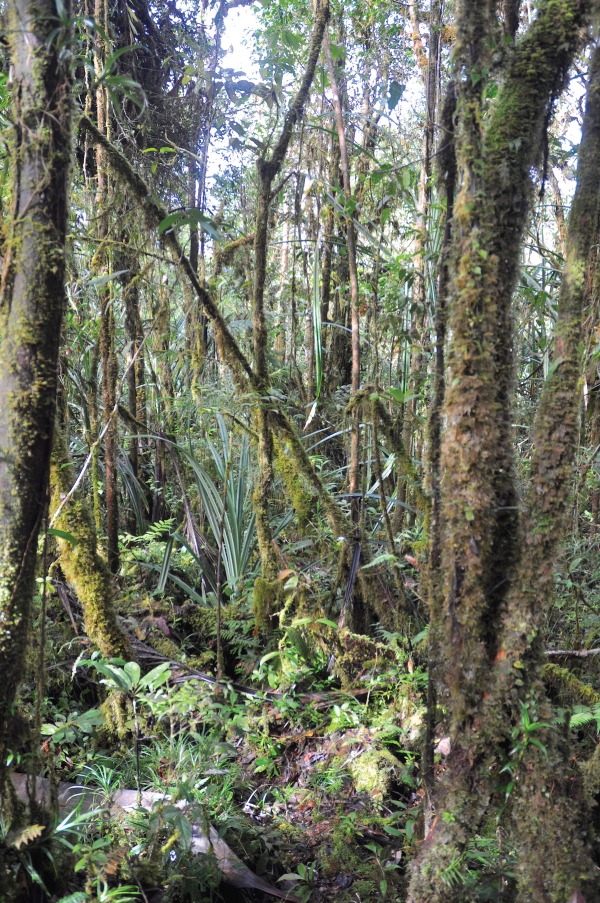
Habitat of *Cyrtodactylus tanim* n. sp.: lower montane forest on karst basement in Western Province, Papua New Guinea. Photograph: Paul M. Oliver.

*Cyrtodactylus tanim* n. sp. was collected in hill forest and lower montane forest where it was quite common, especially at higher elevations. Along a ridge of lower montane forest at approximately 1100 m.a.s.l. (figure 8), up to 10 specimens could reliably be seen over several hours of spotlighting on a single night. They were most commonly seen perched at relatively low heights (less than 3 m above substrate) on limestone faces, or on nearby small trees, roots, or lianas. Two eggs are visible in paratypes (NMV75958 and NMV75959) at varying stages of development.

Above 1000 m.a.s.l. *Cyrtodactylus tanim* n. sp. was the only gecko present, but at the two lower elevation sites (less than 900 m.a.s.l.) it occurred sympatrically with *C. capreoloides* and *C. serratus*. No habitat segregation with the former was obvious. *Cyrtodactylus serratus* appeared to be much rarer (six specimens in three weeks) and was observed at greater heights in the forest strata (e.g. on lianas or in larger forest trees more than 3 m above the ground), suggesting this much larger species is more arboreal than *Cyrtodactylus tanim* n. sp.

#### 3.3.11. Etymology

‘Tanim’, ‘Tanem’ or ‘Tanemkan’ is the ‘tokples’ name that Faiwol speakers from western Papua New Guinea gave specifically for *Cyrtodactylus* geckos, both on this survey, and earlier surveys undertaken by Fred Parker around Wangbin and Migalsimbip Villages in 1969 (personal communication). Incidentally, several local people including experienced hunters, showed distaste for *Cyrtodactylus* geckos, and were reluctant to touch, hold, or in the case of large specimens even look at them.

## 4. Discussion

### 4.1. Ecological diversification, specialization and the exceptional diversification of *Cyrtodactylus*

*Cyrtodactylus* are an exceptionally species-rich lizard clade, currently with over 250 recognized species [53], and that number continues to climb each year [35,54,55]. A key challenge for understanding such large radiations is teasing apart the relative roles that ecological and non-ecological processes have played in generating diversity [56]. However, compared to some other similarly diverse lizard radiations such as *Anolis* and *Liolaemus* [57,58], there has been very little work on the drivers of diversity across *Cyrtodactylus*.

*Cyrtodactylus tanim* n. sp. shows strong evidence of specializations to use complex three-dimensional rock habitats such as karst. It has generally longer limbs, and a flatter head with bigger eyes than similar-sized taxa, including all close non-karst-associated relatives. Larger sample sizes of related taxa, and more accurate measurements based on key skeletal elements would increase the strength of statistical inference, but most Melanesian *Cyrtodactylus* species are rare and housed in collections scattered across the world. Even with this caveat, the observed trends in morphological characters are consistent with background data on how lizard morphology correlates with ecology [21]. Long limbs may widen the centre of gravity and increase their reach in a complex three-dimensional environment [59]. A flatter, narrower head may be linked to accessing tight retreats [12,13]. Larger eyes may relate to using very dark environments such as the interior of caves and overhangs [16]. While most aspects of head size show evidence of reduction relative to body length, orbital width shows the opposite trend, suggesting divergent but strong selection processes. It is also notable that the new species was observed regularly on vegetation. We would argue this does not undermine the argument that it is a karst specialist for two reasons: first, saxicoline specialists often forage on adjacent vegetation, even if they are adapted to rocky microhabitats; second, individuals within complex karst microhabitats are less likely to be detected, leading to potential observational bias towards actively foraging individuals. Indeed, these potential biases emphasize the potential value of morphological data to complement observational data when testing hypotheses of specialization.

The diversification of *Cyrtodactylus* across rock ‘islands’ may also be linked to refugial dynamics [18,19]. There is currently a lack of evidence, however, that climatic refugial processes have played a role in the evolution of *Cyrtodactylus tanim*. This species is partially sympatric with the widespread *Cyrtodactylus capreoloides*, *C. tanim*'s presumed closest relative, and no obvious climatic or rainfall gradients separate these taxa. Dating evidence also suggest it diverged during, not after, the orogeny of the central fold mountains during the late Miocene/early Pliocene [25]. Both patterns are consistent with divergence primarily being linked to ecological opportunity. These data do not rule out the possibility that historical climatic processes may have played an initial role in the isolation and divergence of these taxa, but at this stage there is no evidence to support such a hypothesis.

This study provides the first evidence of specialization to saxicoline microhabitats in New Guinean *Cyrtodactylus*, and as far we can tell the first evidence of specific morphological adaptations to karst in any *Cyrtodactylus* lineage. It also suggests (while we did not measure exactly the same characters) convergence with limb proportions and habitus with cave dwelling *Cyrtodactylus* in Indochina [22]. The New Guinean *Cyrtodactylus* radiation also shows evidence of parallel evolution of gigantism, suggesting ecological release and diversification along another axis [23]. These data provide more evidence for an important role for ecological diversification in the accumulation of *Cyrtodactylus* diversity, especially sympatric taxa, at least in Melanesia. However, studies of morphological diversification in this genus are sparse and geographically localized, and there remain unresolved and untapped opportunities to test for parallelism and convergence, and to better understand the drivers of exceptional diversification. One key challenge to realizing this potential is developing standardized and more accurate approaches (such as CT-scanning and X-rays) to obtaining ecomorphological data.

### 4.2. Karst endemism in New Guinea

Limestone karsts in many tropical and arid regions of the world are home to unique endemic vertebrate taxa [2–4,60]. However *Cyrtodactylus tanim* is the first putatively specialist lizard, and perhaps only the second specialist vertebrate [26], thus far reported from the vast karst areas of New Guinea. We suspect that there are two reasons for this contrasting pattern. First, much of the karst in New Guinea is relatively young, contiguous, and may not have been present through profound aridification events, providing fewer opportunities for persistence of deeply divergent relictual and disjunct endemic lineages. Second, most taxa from New Guinea are very poorly known, and in the absence of studies of functional specialization and adaptation, the number of karst specialists is likely to be underestimated. For instance, a number of frog taxa collected in sympatry with *Cyrtodactylus tanim* are only known from scattered karst in the southern fold mountains, including *Callulops medioclivus* [61], *Choerophryne gracilirostris* [62], *Cophixalus caverniphilis* [63], *Cophixalus wempi* [64] and *Copiula annanoreenae* [65]. Indeed, compared to most other frogs with free-living tadpoles, these direct-developing microhylid species are potentially pre-adapted to heavily dissected karst country with high rainfall but little standing water. Other poorly known frog and lizard taxa are also only known from karst areas elsewhere in New Guinea [66,67]. These data strongly suggest that New Guinea may still be harbouring additional, unrecognized, karst-associated, vertebrate fauna. Further work to better understand the patterns of diversity, specialization and endemism in the large but relatively young karst areas of New Guinea will provide a fascinating contrast against karst biotas elsewhere in the tropics.

## Data accessibility

Additional appendices supporting this article have been uploaded as part of the electronic supplementary material available on Dryad: http://dx.doi.org/10.5061/dryad.48m70 [68]. All new sequence data have been accessioned in GenBank no. MF707373-MF706380.
